# Somatosensory and psychological phenotypes associated with neuropathic pain in entrapment neuropathy

**DOI:** 10.1097/j.pain.0000000000002102

**Published:** 2020-10-09

**Authors:** Luis Matesanz, Andrea C Hausheer, Georgios Baskozos, David L.H. Bennett, Annina B. Schmid

**Affiliations:** a Nuffield Department for Clinical Neurosciences, University of Oxford, Oxford, United Kingdom; b Department of Physical Therapy, Occupational Therapy, Rehabilitation and Physical Medicine, Escuela Internacional de Doctorado, Universidad Rey Juan Carlos, Alcorcón, Spain; c School of Health Professions, Institute of Physiotherapy, Zurich University of Applied Sciences, Winterthur, Switzerland

**Keywords:** Neuropathic pain, Quantitative sensory testing, Entrapment neuropathy, Carpal tunnel syndrome

## Abstract

It currently remains unclear why some patients with entrapment neuropathies develop neuropathic pain (neuP), whereas others have non-neuP, presumably of nociceptive character. Studying patients with carpal tunnel syndrome (CTS), this cross-sectional cohort study investigated changes in somatosensory structure and function as well as emotional well-being specific to the presence and severity of neuP. Patients with CTS (n = 108) were subgrouped by the DN4 questionnaire into those without and with neuP. The latter group was further subdivided into mild and moderate/severe neuP using a pain visual analogue scale. N = 32 participants served as healthy controls. All participants underwent a clinical examination, quantitative sensory testing, electrodiagnostic testing (EDT), and skin biopsy to determine the structural integrity of dermal and intraepidermal nerve fibres. Patients also completed questionnaires evaluating symptom severity and functional deficits, pain distribution, sleep quality, and emotional well-being. The overall prevalence of neuP in patients with CTS was 80%, of which 63% had mild neuP. Symptom severity and functional deficits as well as somatosensory dysfunction was more pronounced with the presence and increasing severity of neuP. No difference was identified among patient groups for EDT and nerve fibre integrity on biopsies. The severity of neuP was accompanied by more pronounced deficits in emotional well-being and sleep quality. Intriguingly, extraterritorial spread of symptoms was more prevalent in patients with moderate/severe neuP, indicating the presence of central mechanisms. NeuP is common in patients with CTS, and its severity is related to the extent of somatosensory dysfunction and a compromise of emotional well-being.

## Abstract 2

Supplemental Digital Content is Available in the Text.

The severity more than the presence of neuropathic pain is related to the extent of neuropathy and a compromise of emotional well-being in entrapment neuropathies.

## 1. Background

Entrapment neuropathies represent the most prevalent peripheral neuropathy and are the most common cause of neuropathic pain (neuP). Carpal tunnel syndrome (CTS) is the most prevalent entrapment neuropathy with a lifetime risk of ∼10% that increases to 85% in patients with diabetes.^48^ Patients mostly experience tingling and numbness in the hand and loss of dexterity. In addition, some patients experience pain, which can impact on their daily functioning.

According to the neuP grading system,^16^ patients with electrodiagnostically confirmed CTS and symptoms in their hand are automatically classed as having at least probable neuP and definite neuP if sensory abnormalities are present. However, the patients' description of their pain sometimes indicates the presence of nociceptive rather than neuP.^31^ Indeed, the use of screening questionnaires revealed that the prevalence of neuP in patients with CTS varies with values reported from 31% to 77%.^21,37,49,50,56^ This together with the absence or at best weak correlation between pain and measures of nerve pathology (eg, nerve conduction studies)^20,51^ has led to the hypothesis that in some patients, pain may originate from structures other than the nerve such as the flexor tendons.^24^

It currently remains unclear why some patients with CTS develop neuP, whereas others do not experience neuP but pain of predominant nociceptive character. One hypothesis is that neuropathy is more severe in patients with neuP compared with those without neuP. However, the evidence for this is currently controversial,^21,37,50^ with most studies reporting no association between electrodiagnostic test severity and the presence of neuP. Of note, electrodiagnostic testing only examines loss of function of large nerve fibres, thus providing only limited information on the potential spectrum of nerve pathology.

A better understanding of the prevalence of neuP and its underlying disease process is crucial to determine risk factors and guide management for these patients. This cross-sectional cohort study provides an in-depth evaluation of the somatosensory phenotype of patients with CTS with and without neuP. We thereby aim to (1) identify changes in the somatosensory structure and function specific to the presence and severity of neuP and (2) explore whether differences in demographic, clinical, and emotional well-being are related to the presence and severity of neuP.

## 2. Methods

### 2.1. Participants

One hundred and eight patients who met electrodiagnostic^1^ and clinical criteria^2^ for CTS participated in the study. Patients were recruited through the neurophysiology and hand surgery departments at Oxford University Hospitals, local print media, and public notice boards. Patients were excluded if electrodiagnostic findings were indicative of other peripheral neuropathies than CTS, if another medical condition affecting the upper extremity or neck was present (eg, tennis elbow or hand osteoarthritis), if a previous history of surgery or trauma to the upper limb or neck existed, or if CTS was caused by pregnancy or diabetes. Proportionally age- and sex-matched healthy controls (n = 32) were recruited through public notice boards and media advertisements. The study was approved by the national ethics committee (London Riverside, Ref 10/H0706/35), and all participants gave informed written consent before participating. Primary publications containing parts of the Oxford CTS cohort have been previously published.^6,46^

### 2.2. Patient subgroups

Patients were divided into those with and without neuP using the DN4 questionnaire.^10^ This questionnaire is composed of questions evaluating a range of sensory descriptors and a short sensory examination. Sensory descriptors include the presence or absence of burning and painfully cold-like pain, electric shocks, tingling, pins and needles, numbness, and itching. The sensory examination evaluates the presence or absence of hypoesthesia to touch, hypoesthesia to pinprick, and brush allodynia. A DN4 score of ≥4 was interpreted as neuP. Because the DN4 was designed to differentiate neuropathic from somatic pain, we interpreted a score of <4 (no neuP) to represent pain of likely nociceptive character. We specifically decided against the use of the neuP grading system,^16^ which has previously been applied to classify patients in similar studies.^22,42,55^ This was particularly important in our cohort, as the grading system would automatically classify all patients with CTS as having at least probable neuP because of the presence of nerve conduction abnormalities. Moreover, our question was focused on neuP vs no neuP (nociceptive pain) rather than painful vs pain-free neuropathies as in previous studies.

To examine the impact of neuP severity on the clinical phenotype, we further classed those with neuP as having mild (<4) or moderate/severe neuP using a cutoff of ≥4 on a visual analogue scale for average pain during the past 24 hours.^55^

### 2.3. Symptom severity, functional deficits, and characteristics of neuropathic pain

The Boston Carpal Tunnel Questionnaire was used to assess symptom severity and functional deficits in patients with CTS.^32^ Characteristics of neuP were evaluated with the Neuropathic Pain Symptom Inventory,^11^ which distinguishes superficial, deep, paroxysmal, or evoked pain as well as paraesthesia and dysaesthesia each on a scale from 0 to 10 with the total score ranging from 0 to 100.^17^ Patients also marked their spread of symptoms on a hand and body diagram. The patterns were dichotomized into median and extramedian spread and the presence or absence of proximal spread of symptoms beyond the hand.^26^ Spread of symptoms outside the distribution of the median nerve has previously been associated with central sensitisation in patients with CTS.^62^

### 2.4. Clinical examination

Light touch and pinprick were tested with cotton wool and a neurotip over the palmar surface of the index fingertip. As sensation may be altered even in hand areas not innervated by the affected median nerve in patients with CTS,^46^ sensation was recorded as normal or reduced compared with the proximal ventral forearm. We further performed 3 commonly used clinical provocation tests: Tinel sign involved light percussion over the median nerve just proximal to the carpal tunnel. A positive test was recorded if characteristic paraesthesia or shooting pain radiating into the fingers was provoked. The Phalen test involved active end of range wrist flexion. A reproduction of symptoms (eg, paraesthesia and numbness) in the median nerve territory within 1 minute was considered a positive test.^39^ For the carpal compression test, moderate pressure was exerted with the investigators' thumbs over the transverse carpal ligament with the wrist in a neutral position.^19^ The test was deemed positive if paraesthesia or numbness was provoked in the median nerve territory of the hand within a maximum period of 30 seconds. Thenar wasting was graded as present or absent. Muscle strength of the abductor pollicis brevis was graded according to the Medical Research Council Manual Muscle Testing scale ranging from M0 to M5, with M3 indicating full range against gravity and M5 indicating activation against the examiner's full resistance with a full range of motion.^35^

### 2.5. Emotional well-being and sleep quality

To evaluate emotional well-being, participants completed the Depression Anxiety and Positive Outlook Scale.^40^ They also completed the 13-item Pain Catastrophizing Scale,^52^ which contains subscales for rumination, magnification, and helplessness, as well as the short form Pain Anxiety Symptoms Scale (PASS-20)^34^ with subscales describing cognitive factors, escape, fear, and physiological anxiety. Sleep disturbance was evaluated with the Insomnia Severity Index, which classifies patients into no insomnia (0-7), subthreshold (8-14), moderate (15-21), or severe insomnia (22-28).^7^

### 2.6. Quantitative sensory testing

Quantitative sensory testing was used to determine somatosensory phenotypes according to the previously published protocol by the German Research Network on Neuropathic Pain.^45^ Cold and warm detection thresholds as well as cold and heat pain thresholds and thermal sensory limen were measured with a ThermoTester (Somedic, Sweden, 25 × 50 mm thermode). We also recorded paradoxical heat sensations during thermal sensory limen testing. Mechanical detection was measured with von Frey hairs and mechanical pain thresholds with weighted pin-prick stimulators. Mechanical pain sensitivity was examined with a numerical pain rating scale (0-100) during 5 sets of 7 pseudorandom pin-prick stimulations. Intermingled with these pin-prick stimulations were 5 sets of 3 light touch stimulations with a cotton wisp, a cotton wool tip, and a standardized brush (Sense-lab) to determine the presence of allodynia. Pressure pain thresholds were evaluated with a manual algometer (Wagner Instruments, Greenwich, CT) and vibration detection threshold with a Rydel–Seiffer tuning fork. The wind-up ratio was measured as the mean numerical pain rating of 5 trains of 10 pin-prick stimuli divided by the mean rating of 5 single stimuli.

The patients were familiarised with the quantitative sensory testing (QST) on the dorsum of the nonexperimental hand followed by testing on the palmar side of the affected index finger (innervated by the median nerve). We also evaluated QST in an extraterritorial area over the dorsum of the hand (innervated by the radial nerve). Whereas the testing area for temperature thresholds was smaller over the index finger (∼10 × 50 mm) than the dorsum of the hand (25 × 50 mm), the areas were comparable between participant groups, hence not influencing our findings. Pressure pain thresholds were recorded over the thenar eminence and brachioradial muscle and vibration detection thresholds over the palmar side of the distal end of the second metacarpal or ulnar styloid for the median and extramedian areas, respectively.

Quantitative sensory testing data (except for cold and heat pain and vibration detection thresholds) were log transformed to achieve normally distributed data.^33,38^ *Z* scores ((value of the participant-mean value of healthy controls)/SD of healthy controls)^45^ were calculated using the proportionally matched healthy control group. A small constant of 0.1 was added to the MPS to avoid loss of zero rating values.^45^

### 2.7. Electrodiagnostic tests

Electrodiagnostic testing (EDT) was performed with an ADVANCE system (Neurometrix) and conventional reusable electrodes. Hand temperature was standardized to >31°C. Sensory orthodromic recordings were made by stimulating the index finger and recording from the wrist. Motor studies were performed by recording from the abductor pollicis brevis stimulated from the wrist and antecubital fossa. To determine the presence of a very mild EDT abnormality, an increased mixed latency of the median sensory nerve action potential compared with ulnar sensory nerve action potential on digit IV stimulation shown by a “double peak” was considered abnormal.^58^ In addition, a difference of >0.4 ms in median vs ulnar motor latency measured over a fixed distance of 8 cm and recorded over the lumbrical and palmar interossei muscles was considered abnormal.^41^ Electrodiagnostic test severity was graded on a scale from 1 (very mild) to 6 (extremely severe) according to previously published criteria.^8^

### 2.8. Skin histology

A 3-mm diameter skin biopsy was taken under subcutaneous anaesthesia on the ventroradial side of the proximal phalanx of the index finger innervated by the median nerve. The biopsy was fixed in fresh periodate-lysine-paraformaldehyde for 30 minutes. The tissue was then washed in phosphate buffer and stored for 2 to 3 days in sucrose in phosphate buffer. After embedding in optimal cutting temperature gel, the tissue was frozen and stored at −80°C. Staining was performed using a previously described free-floating method,^46^ using protein gene product 9.5 (PGP 9.5 Ultraclone, Isle of Wight, United Kingdom, 1:1000; Zytomed, Berlin, Germany 1:200) and myelin basic protein (Abcam, Cambridge, United Kingdom 1:500) as primary antibodies and Cy3 (Stratech, Ely, United Kingdom 1:1000) and Alexa Fluor 488 (Abcam, 1: 500) as secondary antibodies.

Intraepidermal nerve fibre density (IENFD) was quantified in 50-μm skin sections using an Axio LSM 700 microscope with an Observer Z1 imaging system (Zeiss, Cambridge, United Kingdom) by determining the amount of fibres per millimeter epidermis according to current guidelines.^30^ We also quantified dermal innervation by evaluating the number of Meissner corpuscles per millimeter epidermis, the percentage of PGP^+^ dermal nerve bundles containing MBP, and the mean nodal length as previously reported.^46^

### 2.9. Statistical analysis

SPSS Version 27 (IBM) was used for statistical analyses. Normality of data was assessed by visual inspection and using the Shapiro–Wilk test for normality.

Demographic variables, skin histology data, psychological and sleep questionnaires, and EDT parameters were compared among groups (healthy, no neuP, mild neuP, and moderate/severe neuP) with one-way analysis of variance (ANOVA) or Kruskal–Wallis statistics followed by planned contrasts. As we were interested in effects of (1) the presence of neuP and (2) the severity of neuP, we used Helmert contrasts for planned follow-up comparisons. This type of contrast compares each level of our categorical variable “patient group” with the mean of the subsequent levels. As such, the planned contrasts included (1) healthy vs combined patient groups (to confirm differences between patients and healthy controls), (2) no neuP vs combined neuP groups (to evaluate the effect of the presence of neuP), and (3) mild neuP vs moderate/severe neuP (to evaluate the effect of neuP severity). The nonparametric equivalent for the Helmert contrast was used for non-normally distributed data,^47^ and the significance cutoff was adjusted for multiple testing (Bonferroni correction). Symptom and function severity were only evaluated in the 3 patient subgroups using Kruskal–Wallis tests followed by 2 Helmert contrasts (no neuP vs combined neuP groups and mild neuP vs moderate/severe neuP). Findings of the clinical examination and medication intake were compared among groups with chi-square statistics or Fisher exact tests as appropriate. This was followed by 2 planned comparisons, Bonferroni adjusted for multiple testing (no neuP vs combined neuP groups and mild neuP vs moderate/severe neuP), reflecting Helmert contrasts.

Quantitative sensory testing *z* scores were analysed with 4 one-way multivariate ANOVAs (MANOVAs) using the combined QST detection or pain thresholds as the response variables and patient group as the independent variable for both the median and radial territories. Pillai's trace statistics, which is robust to unbalanced designs, is reported. We followed the significant MANOVAs up with one-way univariate ANOVAs followed by Helmert contrasts to test the hypothesis that clinical phenotypes are most pronounced in patients with moderate/severe neuP, followed by those with mild and no neuP, whereas healthy participants show the least deficits.

We also used a recently published algorithm^5,59^ that allocates each patient into 1 of 3 sensory phenotypes: (1) loss of thermal and mechanical detection (“sensory loss”), (2) intact sensory function, often combined with thermal hyperalgesia or allodynia (“thermal hyperalgesia”), and (3) loss of thermal detection, but not mechanical detection, accompanied by mechanical hyperalgesia or allodynia (“mechanical hyperalgesia”).^59^ The deterministic version of the algorithm was used, in which each patient is sorted to 1 phenotype and no mixed phenotypes are possible. Fisher exact tests were used to compare the frequency of QST phenotypes among groups.

## 3. Results

### 3.1. Most patients with carpal tunnel syndrome have neuropathic pain

The demographic data are described in Table 1. Most patients with CTS had likely neuP (80%), whereas 20% were classified as unlikely neuP by the DN4 and therefore presumably have pain of nociceptive character. Of those patients with neuP, 63% were classified as having mild neuP, whereas 37% had moderate/severe neuP. The groups were comparable in regard to age, sex, height, and weight. Duration of symptoms was different among groups (H(2) = 10.1, *P* = 0.006), with Helmert contrasts demonstrating that this was caused by shorter symptom duration in patients with moderate/severe neuP than those with mild neuP.

**Table 1:** Demographic data.

	Healthy	No neuP	Mild neuP	Mod/sev neuP	*P*
No. of participants, n (%)	32	22 (20)	54 (50)	32 (30)	
Sex female (%)	24 (75)	12 (55)	37 (69)	23 (72)	0.426#
Mean age (SD), yrs	57.2 (12.4)	59.0 (14.5)	59.9 (13.5)	57.2 (10.1)	0.725*
Mean height (SD), cm	163.02 (29.9)	168.3 (9.6)	165.7 (10.4)	168.0 (9.0)	0.599*
Mean weight (SD), kg	73.8 (16.9)	75.1 (9.8)	73.0 (17.0)	79.6 (16.8)	0.307*
Median symptom duration [IQR], mo		40 [83]	48 [42]†	18 [30]	0.006*

* *P* values are presented for one-way ANOVAs. # P value represents χ^2^ test association values.Significant overall comparisons are highlighted in bold.

† Significant Bonferroni-adjusted Helmert contrasts are indicated for mild vs moderate/severe neuP groups. Contrasts between no neuP vs combined neuP groups were not significant.

ANOVA, analysis of variance; IQR, interquartile range.; neuP, neuropathic pain; SD, standard deviation

Pain medication to alleviate CTS symptoms was taken by 36% of patients (Supplementary Table 1, available at http://links.lww.com/PAIN/B203). Whereas no differences were apparent between patients with and without neuP, patients with moderate/severe neuP reported more analgesic drug use than those with mild neuP; however, this marginally failed to reach statistical significance. There were no differences among groups for the types of medications used apart from paracetamol and opioids, which were more frequently taken by patients with moderate/severe neuP compared with those with mild neuP.

### 3.2. Symptom severity and functional deficits are more pronounced with the presence and increasing severity of neuropathic pain

Data for symptom severity and functional deficits are summarised in Table 2. Planned contrasts revealed that patients with neuP (combined group) experienced more pronounced symptoms than those with no neuP, except for the deep and evoked pain domain of the NPSI. In addition, symptom severity was consistently higher in patients with moderate/severe neuP compared with those with mild neuP. Similarly, functional deficits measured by the Boston Functional Status Scale were higher in patients with neuP compared with those without neuP.

**Table 2:** Symptom severity, functional deficits, and clinical findings.

	No neuP	Mild neuP	Mod/sev neuP	*P*
Symptom severity, median [IQR]				
Boston Symptom Scale	2.1 [1.1]‡	2.4 [0.9]§	3.2 [0.9]	**<0.0001#**
NPSI total	10.0 [17.5]‡	16.0 [15.8]§	38.5 [20.8]	**<0.0001#**
NPSI burning	0.0 [0.0]‡	0.0 [4.0]	2.0 [6.0]	**0.001#**
NPSI deep	0.0 [2.1]	0.0 [2.0]§	2.5 [4.0]	**0.007#**
NPSI paroxysmal	0.0 [0.1]‡	0.0 [2.1]§	3.0 [4.0]	**<0.0001#**
NPSI evoked	0.0 [0.1]	0.0 [2.0]§	2.7 [3.7]	**<0.0001#**
NPSI paraesthesia	3.5 [3.1]‡	5.3 [5.5]§	7.0 [4.3]	**<0.0001#**
Functional deficits, median [IQR]				
Boston Function Scale	1.6 [0.9]‡	1.9 [0.9]	2.6 [1.4]	**0.004#**
Symptom distribution, n (%)				
Extramedian spread	10 (46)	19 (35)§	21 (66)	0.024*
Proximal spread	11 (50)	31 (57)	23 (72)	0.229*
Clinical examination, n (%) abnormal				
Light touch	1 (5)‡	30 (56)	16 (50)	**0.004**†
Pinprick	6 (27)‡	38 (72)	19 (59)	<0.0001*
Phalen test	13 (62)	37 (82)	24 (80)	0.169*
Tinel sign	7 (33)	23 (46)	20 (67)	0.050*
Compression sign	8 (38)	27 (55)	13 (43)	0.354*
Muscle strength‖				
MRC3	0 (0)	0 (0)	1 (4)	
MRC4	3 (20)	12 (30)	5 (20)	0.617†
MRC5	12 (80)	29 (71)	19 (76)	
Thenar wasting	6 (29)	19 (35)	7 (22)	0.423*

Data are shown as median [IQR] or n (%)

* *P* values reflect χ^2^ associations. #*P* values reflect Kruskal Wallis results.

† *P* values reflect Fisher exact test associations.Significant overall comparisons are highlighted in bold.

‡ Significant Bonferroni-adjusted Helmert contrasts are indicated for no neuP vs combined neuP groups.

§ Significant Bonferroni-adjusted Helmert contrasts are indicated for mild vs moderate/severe neuP groups.

‖ Data for 27 patients not recorded.

IQR, interquartile range; MRC, Medical Research Council Muscle Strength Scale; neuP, neuropathic pain; NPSI, Neuropathic Pain Symptom Inventory.

Patients with moderate/severe neuP had a higher tendency to have extraterritorial symptoms (66%) compared with patients with mild neuP (35%) but not those without neuP (46%, *P* = 0.024).

### 3.3. Clinical examination findings

Patients with neuP exhibited more sensory abnormalities on light touch and pin-prick testing compared with those without neuP (Table 2). The frequencies of motor signs as well as a positive Phalen test and compression sign were comparable among groups. The overall chi-square test for Tinel sign was significant; however, planned contrasts were not significant after Bonferroni correction for the number of planned comparisons.

### 3.4. Somatosensory dysfunction of some parameters is greater in neuropathic pain

**Figure 1.**
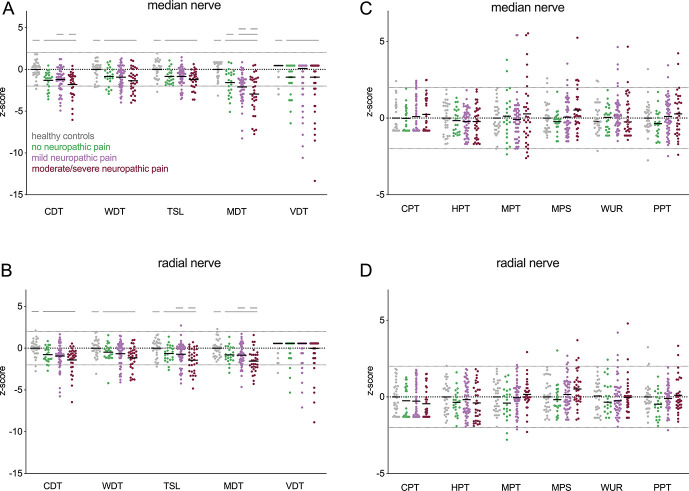
Somatosensory phenotypes as determined with quantitative sensory testing (QST Z-scores): (A) Detection thresholds in the median nerve territory demonstrating a larger deficit for all detection thresholds in patients with carpal tunnel syndrome compared with healthy controls. Patients with moderate/severe neuP have more pronounced CDT deficits compared to patients with mild neuP. All Helmert contrasts were significant for MDT, suggesting that mechanical deficits intensify with the presence and increasing severity of neuP. (B) Detection thresholds in the radial nerve territory demonstrating a larger deficit in CDT, WDT, TSL, and MDT for patients with carpal tunnel syndrome compared with healthy controls. Patients with moderate/severe neuP have a more pronounced deficit in TSL and MDT compared with those with mild neuP. (C) Pain thresholds in the median nerve territory are comparable among groups. (D) Pain thresholds in the radial nerve territory are comparable among groups. Straight lines represent significant Helmert contrasts. CDT, cold detection threshold; CPT, cold pain threshold; HPT, heat pain threshold; MDT, mechanical detection threshold; MPS, mechanical pain sensitivity; MPT, mechanical pain threshold; neuP, neuropathic pain; PPT, pressure pain threshold; TSL, thermal sensory limen; VDT, vibration detection threshold; WDT, warm detection threshold; WUR, wind-up ratio.

Quantitative sensory testing data are shown in Figure 1. The most common somatosensory phenotype in patients with CTS was thermal hyperalgesia (45.4%), followed by sensory loss (33.3%) and mechanical hyperalgesia (21.3%).

In the median nerve territory, the MANOVA for the detection thresholds showed a significant effect (V = 0.32, F(15, 402) = 0.327, *P* < 0.0001). Univariate ANOVAs followed by Helmert contrasts (Fig. 1A) revealed deficits in all detection thresholds in the combined patient groups compared with healthy controls (t(136) > 3.20, *P* < 0.002). Patients with moderate/severe neuP were different from those with mild neuP for cold detection (t(136) = 2.09, *P* = 0.032). Of note, all 3 Helmert contrasts were significant for mechanical detection thresholds in the median nerve territory, indicating that mechanical sensory deficits intensify with the presence and increasing severity of neuP (t(136) > 2.18, *P* < 0.03).

In the radial nerve territory, the MANOVA for detection thresholds was significant (V = 0.24, F(15,402) = 2.36, *P* = 0.003). Univariate ANOVAs followed by Helmert contrasts (Fig. 1B) demonstrated deficits in all detection thresholds except for vibration in the combined patient groups compared with healthy controls (t(136) > 2.98, *P* < 0.003). In addition, patients with moderate/severe neuP had stronger deficits in mechanical detection and thermal sensory limen compared with patients with mild neuP (t(136) > 3.65, *P* < 0.012).

No differences between groups were apparent for pain thresholds in both the median (V = 0.13, F(18,387) = 1.00, *P* = 0.452, Fig. 1C) and radial (V = 0.14, F(18,384) = 1.01, *P* = 0.452, Fig. 1D) territories. Paradoxical heat sensations in the median territory were only experienced by 3 patients, all of which had neuP (2 mild and 1 moderate/severe). In the radial nerve area, 6 participants (3 moderate/severe, 1 mild, 1 no neuP, and 1 healthy control) had paradoxical heat sensations. None of the patients presented with allodynia in either innervation territory.

There was no difference in the proportions of somatosensory profiles among patient subgroups (Fisher exact test, *P* = 0.540, Supplementary Table 2, available at http://links.lww.com/PAIN/B203).

### 3.5. Electrodiagnostic test severity is comparable

There were no differences in electrodiagnostic test severity among patient groups (no neuP median [interquartile range] 3.0 [2.0], mild neuP 3.0 [2.0], and moderate/severe neuP 3.0 [2.0], *P* = 0.744, Supplemental Fig 1, available at http://links.lww.com/PAIN/B203).

Kruskal–Wallis tests showed significant group effects for sensory nerve action potential amplitudes (H(3) = 33.3, *P* < 0.0001), sensory nerve conduction velocities (H(3) = 51.67, *P* < 0.0001), and compound motor latencies (H(3) = 40.57, *P* < 0.0001), but not motor action potential amplitudes for the median nerve (Supplementary Table 3, available at http://links.lww.com/PAIN/B203). Planned contrasts revealed differences between healthy controls and the combined patient groups for all parameters (sensory nerve action potential amplitude: U = 557, z = −5.72, *P* < 0.0001; sensory nerve conduction velocity, U = 102, z = −7.18, *P* < 0.0001; distal motor latencies, U = 3084.5, z = 7.29, *P* < 0.0001; compound motor action potentials, U = 1178, z = −2.73, *P* = 0.006), but no other contrasts were significant (*P* > 0.451).

### 3.6. Nerve structure is comparable

There was an effect of group on IENFD (H(3) = 25.34, *P* < 0.0001), with planned contrasts confirming a reduction in IENFD in patients with CTS compared with healthy controls (U = 755, z = −4.75, *P* < 0.0001, Supplemental Fig. 2, available at http://links.lww.com/PAIN/B203), with no other contrasts being significant (*P* > 0.137, Table 3). No differences were present among groups for dermal measures including density of Meissner corpuscles, dermal nerve bundles containing myelin, or nodal length.

**Table 3:** Histological findings in skin biopsies.

	Healthy	No neuP	Mild neuP	Mod/sev neuP	*P*
IENFD (per mm epidermis)	7.8 [3.5]*	4.0 [3.0]	4.6 [4.1]	3.6 [3.8]	**<0.0001**
Meissner corpuscle density (per mm epidermis)	0.4 [0.4]	0.3 [0.5]	0.3 [0.5]	0.4 [0.6]	0.454
PGP+ bundles containing MBP	1.2 [0.4]	1.1 [0.6]	1.2 [0.3]	1.2 [0.4]	0.621
Nodal length	2.5 [0.9]	2.5 [1.5]	2.5 [1.7]	2.3 [1.0]	0.442

Data are presented as median [IQR]. *P* values reflect Kruskal–Wallis results.Significant overall comparisons are highlighted in bold.

No significant contrasts were found between no neuP vs combined neuP groups and mild vs moderate/severe neuP groups.

* Significant Bonferroni-adjusted Helmert contrasts (*P* < 0.017) are indicated for healthy vs combined patient groups.

IENFD, intraepidermal nerve fibre density; MBP, myelin basic protein; neuP, neuropathic pain; PGP, protein gene product 9.5.

### 3.7. Emotional well-being and sleep quality are more impaired with increasing neuropathic pain severity

**Figure 2.**
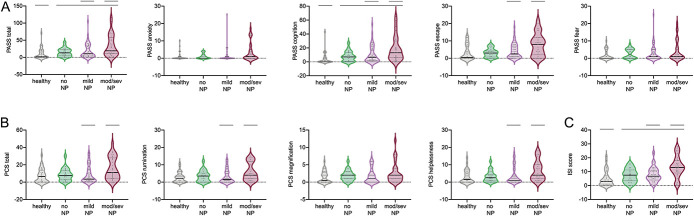
Emotional well-being and sleep quality: (A) The PASS-20 and its subscales demonstrate higher scores for the PASS total and the cognition subscale in patients with carpal tunnel syndrome compared with healthy controls. Patients with moderate/severe neuP have higher scores on PASS total as well as escape and cognition subscores than those with mild neuP. (B) Pain Catastrophizing Scale (PCS) showing mostly higher scores in patients with moderate/severe neuropathic pain compared with those with mild neuP. (C) The Insomnia Severity Index indicates that patients with carpal tunnel syndrome have higher insomnia ratings than healthy controls. Patients with moderate/severe neuP have a more pronounced sleep deficit compared with patients with mild neuP. Violin plots depicting median (solid line), first and third quantiles (dotted lines), and single data points; straight lines represent significant Helmert contrasts. ISI, Insomnia Severity Index; neuP, neuropathic pain; PASS-20, Pain Anxiety Symptoms Scale.

Data of questionnaires evaluating the psychological domain and sleep disturbance are shown in Table 4 and Figure 2.

**Table 4:** Emotional well-being and sleep quality.

	Healthy	No neuP	Mild neuP	Mod/sev neuP	*P*
Insomnia severity	3.0 [6.0]*	6.5 [7.8]	7.0 [7.0]†	13.0 [8.5]	**<0.0001**
PCS total	6.5 [13.0]	7.5 [11.0]	3.5 [13.0]†	11.0 [23.0]	**0.033**
Rumination	2.5 [6.0]	3.5 [5.0]	1.5 [6.0]†	4.0 [10.0]	**0.042**
Magnification	0.5 [3.0]	2.0 [2.0]	1.0 [3.0]	2.0 [3.0]	0.244
Helplessness	1.5 [6.0]	2.5 [4.0]	1.0 [5.0]†	4.0 [8.0]	**0.009**
DAPOS					
Depression	5.0 [2.0]	5.0 [3.0]	6.0 [2.0]	6.0 [6.0]	0.555
Anxiety	3.0 [1.0]	3.0 [2.0]	3.0 [1.0]	3.0 [3.0]	0.572
Outlook	12.5 [4.0]	14.0 [4.0]	12.0 [3.0]	12.0 [5.0]	0.128
PASS-20					
Total	1.5 [18.8]*	13.0 [18.5]	10.5 [29.8]†	19.0 [47.0]	**<0.0001**
Cognition	0.0 [6.0]*	7.0 [13.0]	6.5 [16.0]†	13.0 [24.0]	**<0.0001**
Escape	0.5 [7.0]	3.0 [4.0]	2.5 [6.0]†	8.0 [10.0]	**0.005**
Fear	0.0 [4.0]	2.0 [5.0]	1.0 [5.0]	1.0 [6.0]	0.245
Anxiety	0.0 [1.0]	0.0 [2.0]	0.0 [1.0]	1.0 [5.0]	0.060

Data are shown as median [IQR]. *P* values reflect Kruskal–Wallis test results.Significant overall comparisons are highlighted in bold.

* Significant nonparametric Helmert contrasts Bonferroni adjusted for multiple testing are indicated for healthy vs combined patient groups.

† Significant nonparametric Helmert contrasts Bonferroni adjusted for multiple testing are indicated for mild vs moderate/severe neuP groups. No significant contrasts were apparent between no neuP vs combined neuP groups.

DAPOS, Depression Anxiety and Positive Outlook Scale; IQR, interquartile range; neuP, neuropathic pain; PASS-20, Short Form Pain Anxiety Symptoms Scale; PCS, Pain Catastrophizing Scale.

Whereas no differences among groups were apparent for all domains of the Depression Anxiety and Positive Outlook Scale, significant effects were identified for the PASS-20 total score (H(3) = 18.061, *P* < 0.0001) and the cognitive (H(3) = 23.65, *P* < 0.0001) and escape domains (H(3) = 12.93 m *P* = 0.005). Planned contrasts confirmed higher ratings in patients with CTS compared with healthy controls in 2 PASS-20 measures (total U = 2379, z = 3.35, *P* = 0.01; cognitive U = 2509.5, z = 4.02, *P* < 0.0001). In addition, patients with moderate/severe neuP had higher ratings than patients with mild neuP in several PASS-20 domains (total U = 1125, z = 2.52, *P* = 0.012; cognition U = 1124.5, z = 2.51, *P* = 0.01; escape U = 1114.5, z = 2.44, *P* = 0.015), indicating a stronger compromise in emotional well-being with increasing neuP severity. No other planned contrasts were significant.

There was also an effect of group on the PCS (total score H(3) = 8.72, *P* = 0.033; rumination H(3) = 8.20, *P* = 0.042; helplessness H(3) = 11.64, *P* = 0.009), with more severe rumination (U = 1114.5, z = 2.45, *P* = 0.014), helplessness (U = 1160, z = 2.86, *P* = 0.004), and total PCS scores (U = 1130, z = 2.57, *P* = 0.10) in patients with moderate/severe neuP compared with those with mild neuP, although the other contrasts were not significant.

For the Insomnia Severity Index (H(3) = 26.24, *P* < 0.0001), a more pronounced sleep disturbance was apparent in the combined patient groups compared with healthy controls (U = 2478, z = 3.839, *P* < 0.0001) and in patients with moderate/severe neuP compared with those with mild neuP (U = 1222.5, z = 3.402, *P* = 0.001), indicating more pronounced sleep difficulty with increasing intensity of neuP.

## 4. Discussion

In our cohort of patients with CTS, 20% have no neuP, 50% have mild neuP, and 30% have moderate/severe neuP. The presence of neuP was associated with increased symptom severity and functional deficits as well as deficits in bedside sensory testing. Apart from a more pronounced deficit in mechanical detection, somatosensory profiles were largely comparable among patients with and without neuP. However, an increasing neuP severity was associated with more pronounced loss of function deficits in both the median and radial nerve territories. By contrast, no differences were identified in neurophysiological variables or structural nerve fibre integrity in skin biopsies among patient groups. Notably, many aspects of emotional well-being (eg, PCS rumination and helplessness, as well as PASS cognition and escape) and sleep were more affected with increasing neuP severity. Our findings indicate that apart from clear differences in symptom severity and function deficits, structural and functional somatosensory measures are largely comparable in patients with and without neuP. The severity of neuP is associated with somatosensory nerve dysfunction, but not structural nerve integrity. Of note, an increasing severity of neuP was accompanied by reduced emotional well-being, increased sleep disturbance, and the presence of extraterritorial symptoms, indicating a more dominant contribution of central mechanisms.

The reported prevalence of neuP in patients with CTS varies substantially (31%-77%).^21,49,50,54^ This is most likely attributable to the different screening tools used to detect neuP, none of which has been validated in patients with CTS. In the absence of a validated screening tool for patients with CTS, we decided to use the DN4. Unlike the painDETECT, it focuses on the number of neuropathic features rather than their severity, which is often low in patients with CTS and may thus underestimate the prevalence of neuP. Furthermore, the painDETECT was originally developed for spinally referred leg pain,^18^ whereas the DN4 was validated in a mixed group of nerve disorders,^10^ thus increasing its generalisability. The here-identified 80% of patients having neuP is comparable with other studies that also used the DN4 tool in patients with CTS (65%-77%).^37,56^ The sample of convenience used in our study does not allow inferences about the general prevalence of neuP in CTS. Nevertheless, our data suggest that although most patients have neuP, a significant proportion has non-neuP, presumably of nociceptive origin.

Compared with healthy controls, patients with CTS show loss of function to thermal and mechanical stimuli in the median nerve innervation territory independent of the presence of neuP. This represents the characteristic dysfunction of both small and large nerve fibers as previously reported in CTS^29,46^ and other focal and systemic peripheral neuropathies.^22,42,54,55,57^ Although somatosensory function was largely comparable between patients with and without neuP except for mechanical detection, the increasing severity of neuP was associated with a mechanical and thermal loss of function phenotype. This progressive loss of function phenotype with increasing neuP severity is in line with previous reports in patients with focal and systemic peripheral nerve injuries^22,42,55^ and has been interpreted as an indication that increasing neuP severity is associated with a more pronounced neuropathy. Intriguingly and consistent with previous reports from systemic polyneuropathies,^42,55^ changes in nerve fibre integrity in the skin or the extent of neurophysiological changes were not associated with the presence or severity of neuP.

Extramedian but not proximal spread of symptoms was more common in patients with moderate/severe neuP. Such extramedian spread of symptoms has previously been shown to be associated with extramedian mechanical and thermal hyperalgesia^62^ and has thus been attributed to central mechanisms.^62,63^ In addition, we found a more pronounced hypoaesthesia in the radial nerve territory in patients with moderate/severe neuP compared with those with mild neuP. We have previously reported such extramedian hyposensitivity in a smaller cohort of patients with CTS.^46^ Although widespread hyperalgesia is commonly accepted as a sign of central mechanisms, hyposensitivity as a sign of nerve dysfunction is usually expected to be restricted to the area of the affected nerve. There is, however, growing evidence that sensory loss can also be found in unaffected areas in patients with neuP.^23,27,28,53,60,61^ In such instances, the extraterritorial sensory loss has been attributed to centrally mediated mechanisms, for instance, to the suppression of normal sensitivity by ongoing pain.^60^ Taken together, our data suggest that central mechanisms are more prominent in patients with moderate/severe neuP. More pronounced central mechanisms may thus be an alternative interpretation to an increased neuropathy severity in driving symptoms in patients with more severe neuP.

Patients with neuP had more pronounced symptom severity and functional deficits than patients without neuP throughout a range of questionnaires. This is in line with previous reports in a range of chronic pain conditions.^4^ As expected due to the neuP subgroup allocation being governed by symptom severity, increasing neuP severity was associated with more pronounced symptoms, but this was also the case for functional deficits. In addition, emotional well-being and sleep impairment was more compromised with increasing neuP severity. These results are in line with previous reports of patients with CTS^37^ and other peripheral neuropathies.^4,22,42,55^ Nevertheless, the average ratings in our cohort were low. Also, it remains unanswered whether the deficits in emotional well-being are a consequence of or a risk factor for more severe neuP. The previously reported decrease of depressive symptoms after carpal tunnel decompression and its correlation with symptom resolution^13^ suggests that depression may be secondary to CTS. This is further corroborated in our own prospective data, which confirm improvements in most emotional well-being parameters after carpal tunnel decompression (Supplementary Table 4, available at http://links.lww.com/PAIN/B203).

Some limitations of this study need to be considered. Our study is a post hoc analysis of 2 published cohorts of exploratory character and did therefore not include an a priori sample size calculation. Nevertheless, our study contains the largest deeply phenotyped CTS cohort to date, and its size was large enough to detect moderate effect sizes among patient groups. However, numbers in some patient subgroups were relatively low. This may have contributed to the absence of group differences for instance in the planned contrasts of neuP and no neuP groups. Another limitation to consider is that analgesic intake was not stopped before somatosensory profiling and may thus have influenced our readings, particularly related to hyperalgesia.

### 4.1. Clinical implications

Although it is clear that there are a proportion of patients with CTS who do not experience neuP (20%), most patients do. Treatment for patients with CTS is currently not stratified for the presence of neuP. Our data suggest that particularly patients with moderate/severe neuP have a distinct phenotype characterised by a more pronounced and widespread somatosensory dysfunction and exacerbated deficits in emotional well-being and sleep quality. Given the excessive wait times for carpal tunnel surgery^3^ and the detrimental effects of poor emotional well-being and sleep quality on general health and quality of life,^12,44^ these patients may need to be prioritised. Indeed, in our cohort that was mostly recruited from surgery waitlists, symptom duration was over two-fold shorter in the moderate/severe subgroup, potentially reflecting an earlier escalation to surgery.

Although surgical decompression is successful in around 75% of patients,^9^ nonsurgical management including pharmacological options remains first-line treatment.^43^ Current guidelines recommend corticosteroid injections but not oral nonsteroidal anti-inflammatory drugs without mentioning neuP drugs.^36^ In our cohort, patients with moderate/severe neuP took more paracetamol and opioids, which are not first-line pharmacological options for neuP.^14^ It could be argued that patients with moderate/severe neuP may benefit from specific neuP drugs, which often target central mechanisms that seemed common in that group. However, trials into neuP medications such as gabapentin for patients with CTS show controversial results.^15,25^ Future studies are required to determine whether stratification by the neuP phenotype may lead to more promising effects of these medications for patients with CTS and whether the risk/benefit of neuP medications outweighs that of surgery.

Of note, our results suggest that the routine diagnostic tests for CTS (Phalen test, Tinel sign, carpal compression test, and electrodiagnostic tests) are not able to identify the presence of neuP. Therefore, simple screening tools such as the DN4 will facilitate the identification of patients who are more severely affected by neuP and may help guide management.

### 4.2. Conclusions

Our cohort has shown that neuP is common in patients with CTS and its presence is accompanied by more severe symptom and function deficits. Apart from a deficit in mechanical detection, the presence of neuP was not associated with substantial changes in somatosensory function or structural nerve pathology. The severity of neuP was accompanied by a more pronounced somatosensory dysfunction. Of note, neuP severity was related to more pronounced deficits in emotional well-being and sleep quality and the presence of extraterritorial spread of symptoms suggesting a more dominant contribution of central mechanisms. These differences between subgroups raise the question whether treatment stratification may help improve management for patients with CTS.

## Conflict of interest statement

D.L.H. Bennett has acted as a consultant on behalf of Oxford Innovation in the past 2 years for Amgen, CODA therapeutic, Bristows, Lilly, Mundipharma, and Theranexus. The remaining authors have no conflicts of interest to declare.

## Appendix A. Supplemental digital content

Supplemental digital content associated with this article can be found online at http://links.lww.com/PAIN/B203.
